# SGLT2 inhibitors: cardiorenal metabolic drugs for the ages

**DOI:** 10.1172/JCI177625

**Published:** 2024-03-01

**Authors:** Ralph A. DeFronzo

**Affiliations:** Diabetes Division, Department of Medicine, University of Texas Health Science Center, San Antonio, Texas, USA.

## When does the story start?

**Figure 1:**
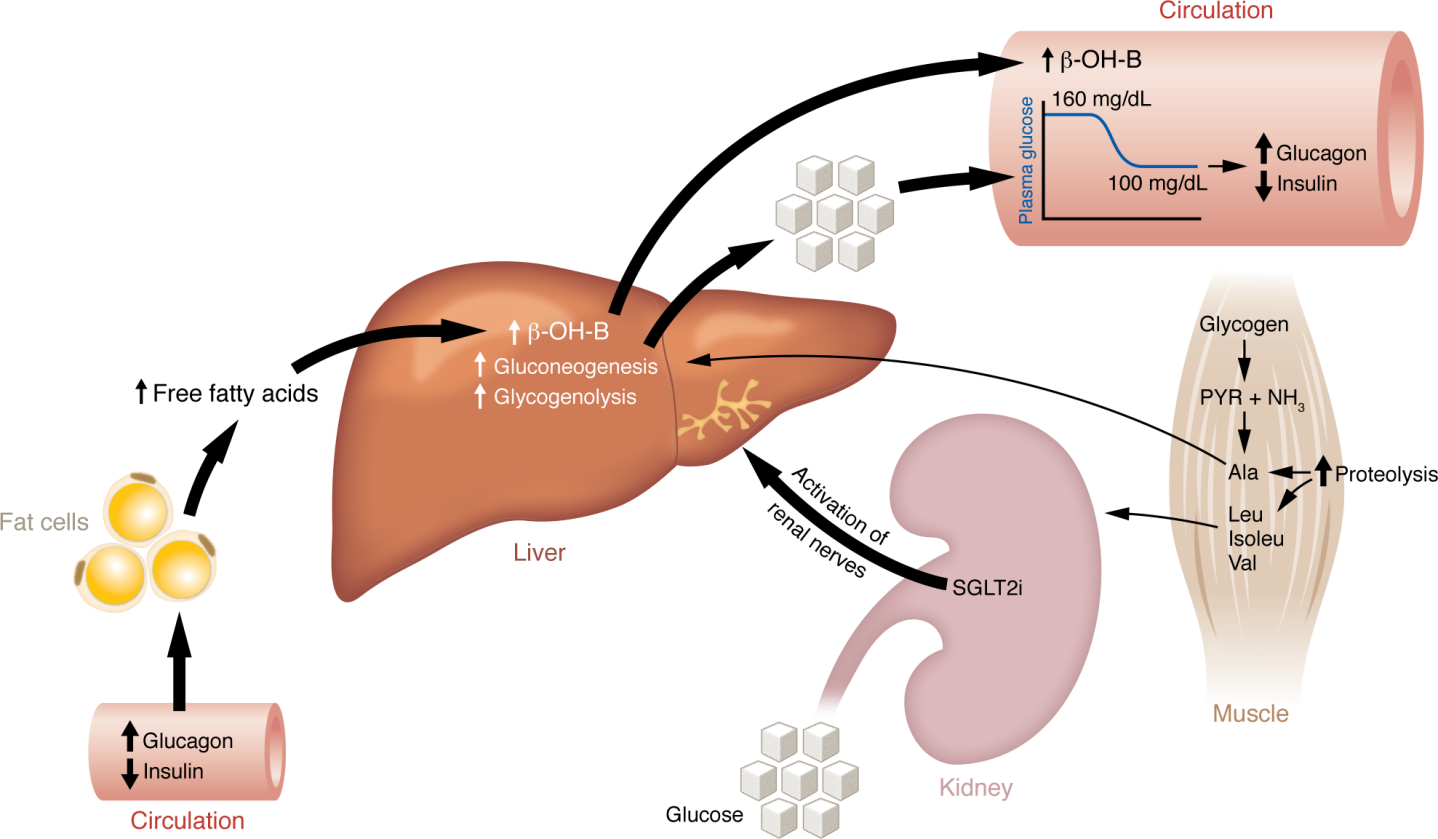
SGLT2 inhibitors acutely induce glucosuria, which persists as long as the SGLT2i therapy is continued. Treatment with SGLT2 inhibitors, such as phlorizin, promotes glucosuria and leads to a decline in fasting plasma glucose concentration, i.e., from 160 to 100 mg/dL. When the fasting plasma glucose declines toward normoglycemic levels, activation of the renal sympathetic nerves stimulates the liver to undergo glucogenesis and glycogenolysis, and quantitatively this increases hepatic glucose production (HGP), matching the glucose amount excreted in the urine, thus preventing hypoglycemia. The decline in plasma glucose concentration creates a state of tissue energy deprivation and leads to a decrease in plasma insulin and an increase in plasma glucagon concentration. These resultant hormonal changes stimulate lipolysis, thus providing substrate for ketogenesis, and reset the liver into the ketogenic mode. Subsequent ketone (β-hydroxybutyrate [β-OH-B]) production provides a fuel-efficient substrate for the heart and the kidney. In muscle, glycogen breakdown is stimulated, providing the carbon skeleton for the synthesis of alanine (Ala), which is released into the circulation and transported to the liver to support gluconeogenesis (known as the alanine cycle). Stimulation of muscle proteolysis also results in the release of alanine as well as other amino acids, in particular the branched-chain amino acids (BCAAs): leucine (Leu); isoleucine (Isoleu); and valine (Val). The liver does not possess BCAA transferase, allowing the BCAAs to be taken up by the heart and kidney where they provide substrate to compensate for the reduction in plasma glucose concentration that occurs secondary to the glucosuric effect of the SGLT2i. PYR, pyruvate.

Having trained as an endocrinologist and diabetologist, as well as a nephrologist interested in phosphate and glucose transport in the kidney (1), it was logical to combine the two disciplines. It also made sense to utilize phlorizin, a dual SGLT2/SGLT1 inhibitor, as a therapeutic option for the treatment of type 2 diabetes (T2D) based on the premise that the induction of glucosuria would reduce the plasma glucose concentration (Figure 1). Because reduced glucose levels ameliorate glucotoxicity, lead to reversal of insulin resistance in muscle (2), and improve β cell function (3), we set out to test phlorizin using partially (90%) pancreatectomized diabetic rats.

## Reversing glucotoxicity

In partially pancreatectomized diabetic rats, insulin sensitivity, assessed with the gold standard euglycemic insulin clamp in awake, unstressed, chronically catheterized rats, was reduced by 30%–40%. In diabetic animals treated with phlorizin by subcutaneous injection for four weeks, whole-body insulin sensitivity, which primarily reflects that of the muscle, was completely normalized. The treatment also normalized the fasting and postmeal plasma glucose concentrations (2, 3). Discontinuation of phlorizin in diabetic rats reverted them into a state of insulin resistance with a diabetic plasma glucose profile. These studies unequivocally established the important role of glucotoxicity in the development of muscle insulin resistance. Thus, they showed that an initial defect in insulin secretion leading to hyperglycemia can result in the development of skeletal muscle insulin resistance. The experiments revealed that phlorizin, a drug that inhibits renal tubular glucose absorption but has no other known metabolic effects, had the capacity to completely reverse insulin resistance and restore normal glucose tolerance. Notably, the induction of glycosuria, with normalization of the plasma glucose profile, could reverse glucotoxicity, and this finding provided definitive in vivo proof for the glucotoxicity hypothesis (2, 3).

To examine the cellular mechanisms via which glucotoxicity induced insulin resistance, we examined in vivo glucose transport and whole-body insulin sensitivity in diabetic rats (4). As previously demonstrated (2), partial pancreatectomy resulted in severe insulin resistance and a parallel 50% decrease in insulin-stimulated glucose transport in adipocytes (4). Levels of GLUT4, measured by immunoblotting, were decreased in adipose cell subcellular membrane fractions, as were the corresponding mRNA levels, as assessed by Northern blotting. Restoration of normoglycemia with phlorizin restored insulin-stimulated glucose transport in adipocytes and insulin-mediated glucose disposal in vivo. However, GLUT4 protein levels within low-density microsomes in the basal and insulin-stimulated state remained reduced by 40%–50%. These studies demonstrated for the first time that (a) changes in the ambient glucose plasma concentration, independent of ambient insulin, can regulate the glucose transport response to insulin without changes in GLUT4 translocation to the plasma membrane and (b) changes in intrinsic GLUT4 functional activity play an important role in the enhanced insulin sensitivity observed with the reversal of glucotoxicity (2, 4).

The effects of chronic hyperglycemia on in vivo insulin secretion were examined after rats underwent 90% pancreatectomy, leaving intact the 10% of the pancreas between the common bile duct and the duodenal sweep (3). Notably, insulin secretion expressed within the remaining percentage of pancreas should be normal unless some acquired defect in β cell function develops. The hyperglycemic clamp technique in awake, unstressed, 90% pancreatectomized diabetic rats revealed that first- and second-phase insulin secretion were severely impaired when expressed per residual pancreas. Furthermore, both phases of insulin secretion completely returned to normal and plasma-glucose concentration normalized following treatment with phlorizin. These results provided conclusive evidence that chronic hyperglycemia impairs insulin secretion and showed that the reversal of glucotoxicity with phlorizin can restore normal β cell function (3).

## From rodents to humans

Based upon these three landmark studies (2–4) in rodents, we approached Bristol-Myers Squibb/AstraZeneca and developed a clinical investigation program to examine the effect of inhibition of renal tubular glucose transport on glucose homeostasis, insulin sensitivity, and insulin secretion in patients with T2D (5). The first of these human studies was published in the *JCI* in 2014. In this double-blind placebo-controlled study (5), patients with insulin-resistant T2D were randomized to treatment with dapagliflozin (10 mg/d) or placebo for 14 days. Dapagliflozin induced glucosuria (75–91 g/d), markedly lowered the fasting plasma glucose concentration, and increased insulin-stimulated whole-body glucose disposal using the euglycemic insulin clamp technique. This study was the first to document an increase in insulin sensitivity following improvement in glycemic control with SGLT2 inhibition. This study also was the first to demonstrate that SGLT2 inhibitor (SGLT2i) administration stimulated endogenous glucose production (EGP) in patients with T2D. Of note, the increase in EGP was associated with an increase in plasma glucagon concentration and a decline in plasma insulin concentration, and the increase in EGP offset by approximately 50% the amount of glucose excreted in the urine (5–7), thereby blunting the decrease in fasting plasma glucose concentration. In another paper published in the *JCI*, Ferrannini and colleagues (8) used a dual-tracer technique to demonstrated that empagliflozin reduced the fasting and postmeal plasma glucose concentration, augmented insulin sensitivity, and stimulated basal EGP. In follow-up studies in patients with T2D, we examined β cell function more intensively using the oral glucose tolerance test (OGTT) and euglycemic insulin clamp (9, 10). Fasting and post-OGTT plasma glucose concentrations declined by 33 and 73 mg/dL, respectively; insulin secretion (Δ C-Pep_0–120_/Δ G_0–120_) increased 2-fold; and β cell function (disposition index) (Δ C-Pep_0–120_/Δ G_0–120_/insulin resistance) rose 2.4-fold, where C-Pep is C-Peptide and G is glucose. These findings document the deleterious impact of glucotoxicity on β cell function in patients with T2D and the improvement in β cell function following amelioration of glucotoxicity by the glucosuric effect produced by SGLT2 inhibition. Of note, a similar beneficial effect of SGLT2i on β cell function was observed by Ferrannini et al. following a meal tolerance test (8).

To examine the mechanism via which SGLT2i stimulated basal EGP, individuals with T2D received dapagliflozin under conditions in which the plasma insulin, glucagon, and glucose concentrations were clamped at the fasting level alone or in combination via the pancreatic clamp technique (11). In the absence of any change in plasma insulin, glucagon, or glucose concentrations, SGLT2i still caused a stimulation of EGP. To further pursue the mechanism responsible for the SGLT2i-induced rise in EGP, studies were performed in individuals with end-stage polycystic kidney disease who had undergone a renal transplant and in whom both native kidneys had been removed (12). When dapagliflozin was administered to these individuals, the stimulation of EGP was markedly blunted, indicating an important role for the renal nerves in mediating the SGLT2i-induced stimulation of EGP. This observation is consistent with the rapid stimulation of EGP, within 15–30 minutes after administration of an SGLT2 inhibitor (13), and uncovers a previously unrecognized renal-hepatic axis. Moreover, this stimulatory effect of SGLT2i on EGP persisted for at least 4 months (13). Furthermore, in normal glucose-tolerant individuals the increase in EGP quantitatively matches the increase in urinary glucose production, such that the fasting plasma glucose remains constant at the basal level (14, 15). This finding explains why SGLT2is do not promote hypoglycemia. Although, at the time these studies were performed, it was unknown whether the site responsible for the increase in EGP was the liver or kidney, follow-up studies combining arterial cannulation with renal vein catheterization and 3-^3^H-glucose and PAH infusion conclusively demonstrated that all of the increase in EGP is derived from the liver (16).

Based upon the established role of deranged tubuloglomerular feedback leading to increased intraglomerular pressure and diabetic nephropathy (17), we predicted that inhibition of proximal sodium along with glucose reabsorption with SGLT2i would enhance sodium delivery to the macular densa cells of the juxtaglomerular apparatus, thereby reducing intraglomerular pressure and slowing the rate of decline in glomerular filtration rate in patients with established diabetic nephropathy (6, 18). Not surprisingly, this prediction was confirmed in multiple prospective studies (6, 19). The effect of SGLT2i therapy to prevent heart failure and reduce cardiovascular mortality in patients with diabetes and established cardiovascular disease and/or risk factors for cardiovascular disease was totally unpredictable (20).

## Conclusions

Who would have thought that studies initiated to control glycemia by reversing glucotoxicity in diabetic rodent models (2–4) and in people with T2D (5) would have eventuated in (a) the discovery of a previously unrecognized renal-hepatic axis involved in the regulation of glucose metabolism (Figure 1); (b) a therapeutic intervention providing both cardiovascular and renal protection; and (c) sustained improvement in glycemic control in patients with T2D? These findings lend credence to the adage that “an apple per day keeps the cardiologist and nephrologist away.”

## Version 1 03/01/2024

Electronic publication
